# Total cholesterol mediates the association between history of gestational diabetes mellitus and bone mineral density in US women aged 20–49 years

**DOI:** 10.1186/s12889-023-17609-0

**Published:** 2024-01-03

**Authors:** Yan Zeng, Li Yin, Xiaoping Yin, Danqing Zhao

**Affiliations:** 1 Department of Obstetrics and Gynecology, Affiliated Hospital of Guizhou Medical University, No.16 Beijing Road, 550004 Guiyang City, People’s Republic of China; 2 Guiyang Maternal and Child Health Care Hospital, Guiyang Children’s Hospital, Guizhou Medical University, 550025 Guiyang, People’s Republic of China

**Keywords:** History of gestational diabetes mellitus, Bone mineral density, Serum total cholesterol, Mediation effect, Premenopausal women

## Abstract

**Objective:**

The aim of this study is to investigate the potential association between a history of gestational diabetes mellitus (GDM) and lumbar bone mineral density (BMD) among premenopausal women, with an additional examination of the mediating role of serum total cholesterol (TC).

**Methods:**

In this cross-sectional study, 1809 women aged 20–49 years with at least one live birth between 2011 and 2018, drawn from the NHANES dataset, were analyzed. GDM history was identified through questionnaires. Using weighted multiple linear regression, we assessed the relationship between GDM history and lumbar BMD. Additionally, mediation analysis was performed to investigate the potential mediating role of TC.

**Results:**

The fully adjusted linear regression model revealed a negative association between a history of GDM and lumbar BMD, indicating a reduction in lumbar BMD (β = -0.023, 95% CI: -0.043, -0.003, *P* = 0.0275). Subgroup analysis highlighted a more pronounced trend in individuals aged ≥ 35 years and with a body mass index ≥ 30 kg/m². Furthermore, mediation analysis demonstrated a significant direct effect of a history of GDM on lumbar BMD (*P* < 0.0001), with serum TC playing a partial mediating role in this interaction (5.33%, *P* = 0.028).

**Conclusions:**

In women aged 20–49 years within the United States, a history of GDM was associated with diminished lumbar BMD, potentially mediated through serum TC.

## Introduction

Gestational diabetes mellitus (GDM), often characterized by abnormal glucose tolerance that emerges or is initially identified during pregnancy [1], stands as a prevalent medical complication encountered during gestation. The prevalence of GDM within the Hyperglycemia and Adverse Pregnancy Outcomes (HAPO) study cohort, utilizing the diagnostic criteria endorsed by the International Association of the Diabetes and Pregnancy Study Groups (IADPSG), varied between 9.3% and 25.5% [2]. The age-adjusted prevalence of a history of GDM was 7.6% in this U.S. women’s sample, representative of the years 2007 to 2014 [3]. GDM is associated with obstetric and neonatal complications, as well as persistent effects on maternal and offspring health. Notably, individuals with a history of GDM exhibit an elevated predisposition to the development of type 2 diabetes mellitus (T2DM), obesity, dyslipidemia, and cardiovascular disease [4–8].

Bone mineral density (BMD) serves as a valuable parameter for osteoporosis assessment [9], and the enduring interest in understanding the relationship between chronic diseases and bone health in women remains a focus of investigation. T2DM has been recognized as one of the risk factors for osteoporotic fractures. Prolonged duration of T2DM, impaired glucose regulation, and persistence of inflammation have been reported to contribute to alterations in BMD related to diabetes [10, 11]. In addition, a cohort study identified a correlation between dyslipidemia and BMD in women, emphasizing a substantial inverse relationship between overall cholesterol levels and lumbar BMD [12]. Given the long-term impact of GDM on women’s health, recognizing the association between a history of GDM and BMD holds significance for predicting and preventing osteoporosis. A Norfolk cohort analysis based on the European Prospective Investigation into Cancer (EPIC-Norfolk) study of people aged 39–79 years showed that a history of GDM increased the risk of hip fracture and falls, and that reduced bone mineral density may be responsible for the increased risk of fracture in women with a history of GDM [13].

Postmenopausal women are acknowledged as being at heightened risk for osteoporosis, and a history of GDM is linked to an increased osteoporotic risk in this group [14]. Under the influence of estrogen deficiency in postmenopausal women, discerning a plausible association between a history of GDM, lipid profile, and bone mineral density remains challenging. However, there are few investigations of bone health in premenopausal women with a history of GDM. Overall, the average age of natural menopause for women in the United States from 2015 to 2018 was 49.9 years [15]. Therefore, we explored the relationship between historical instances of GDM and lumbar BMD in women aged 20–49 years based on samples collected from the National Health and Nutrition Examination Surveys (NHANES) from 2011 to 2018 and further analyzed to quantify the role of serum total cholesterol (TC) in mediating the relationship between a history of GDM and lumbar BMD.

## Materials and methods

### Study population and design

The NHANES serves as a comprehensive cross-sectional study of national representation, meticulously crafted to evaluate the health and nutritional profiles of both adult and pediatric populations within the United States [16]. The survey encompasses queries related to demographics, socioeconomic status, dietary habits, and health-related aspects. The survey’s structure, methodologies, and resultant data are accessible to the public. The protocols governing the NHANES receive endorsement from the National Center for Health Statistics, an entity functioning within the Centers for Disease Control and Prevention. In this context, the engagement of all participants mandates their conscientious and well-informed agreement.

**Fig. 1:**
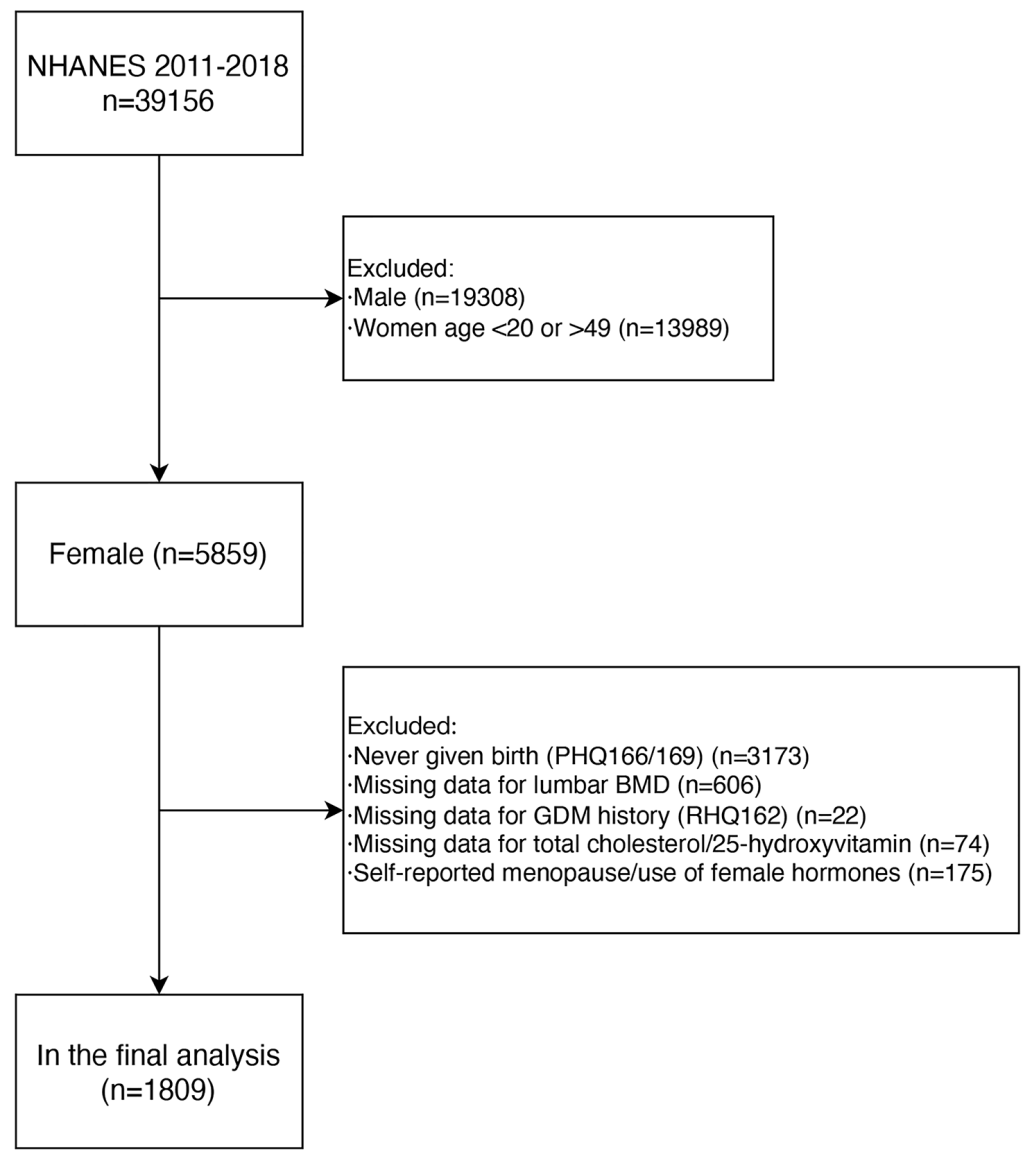
Flowchart of participants selection from the NHANES 2011–2018. NHANES, National Health and Nutrition Examination Survey; BMD, bone mineral density; GDM, gestational diabetes mellitus

In the 2011–2018 NHANES study, a total of 39,156 individuals participated, including a female cohort of 19,848. Following the application of specific exclusion criteria, the final study cohort comprised 1809 participants, as visually demonstrated in Fig. 1.

### A history of GDM, assessment of lumbar BMD and measurement of TC

The exposure for the analysis was the response to the reproductive health questionnaire question RHQ162, women affirming this query were categorized as possessing a GDM history.

The outcome of the present study is lumbar BMD. As a clinical metric, lumbar BMD measurement stands pivotal in the assessment and management of osteoporosis [17]. BMD measurements within NHANES were obtained using dual-energy X-ray absorptiometry (DXA) scanning, which is the most widely accepted method. DXA scans in the NHANES program were performed using a Hologic Discovery Model A densitometer (Hologic, Inc., Bedford, Massachusetts), excluding participants who were pregnant or had a history of radiographic (barium) use within the past 7 days or whose body size exceeded the DXA table limit. Testing was performed by trained and certified radiologic technologists. Data were analyzed using Hologic APEX (version 4.0) software. The detailed information on the DXA inspection program were available on the NHANES website.

Following an overnight fast lasting 8–12 h, venous blood specimens were obtained in the early morning for the measurement of TC. The collected serum specimens underwent processing, storage, and were subsequently shipped to the University of Minnesota in Minneapolis for analysis. Total cholesterol measurement was carried out utilizing an enzymatic method on a Roche/Hitachi Cobas 6000 analyzer.

### Covariates

Data were acquired through questionnaires, physical examinations, and laboratory tests. Standardized questionnaires provided insights into the demographic traits of each participant, encompassing including age, race, education, poverty-to-income ratio (PIR), alcohol consumption, smoking status, history of hypertension, history of diabetes mellitus, as well as a history of delivering an infant weighing 9 pounds or more. The body mass index (BMI) data were extracted from the extensive array of metrics derived from thorough physical examinations. Additionally, concentrations of 25-hydroxyvitamin D (25OHD) were measured from laboratory analysis of blood samples.

### Statistical analysis

Participants were divided into two groups based on their history of GDM. Categorical variables are expressed in terms of numbers (percentage), whereas continuous variables are presented as means and standard deviations (mean ± SD). Profiling the distribution patterns of continuous variables, stratified according to diverse participant characteristics, involved the implementation of either the Wilcoxon rank sum test or the Kruskal-Wallis test. Assessment of statistical significance for categorical variables was executed using chi-square tests. Utilization of weighted multiple linear regression models served as the foundation for analyzing the relationship between GDM history and lumbar BMD. Furthermore, the association between GDM history and TC was also explored, as well as between TC and lumbar BMD. Supplementary stratified analyses were executed, classifying participants based on distinct characteristics such as age, race, BMI, and the occurrence of delivering a baby weighing 9 pounds or more. Three models were assessed: (1) Model I: adjusted none; (2) Model II: adjusted for age and race data only; and (3) Model III: covariate adjustments encompassed age; race; education level; PIR; BMI; diabetes history; hypertension history; alcohol use; smoking; 25OHD and a history of delivering an infant weighing 9 pounds or more.

To evaluate the degree of mediation exerted by TC in the association between GDM history and lumbar BMD, causal mediator analyses were undertaken. We utilized the “mediator” R software package to quantify direct, mediation, and total effects. It’s imperative that mediators exhibit associations with both the exposure and the outcome [18]. Within our investigation, the independent, outcome, and mediating variables were respectively represented by GDM history, lumbar BMD, and TC. This methodology aligns with a causal mediator analysis framework, serving to dissect the comprehensive impact of GDM history into a direct influence on lumbar BMD and an intermediary influence mediated by TC [19]. The PROCESS program facilitated the mediation analyses, employing 5,000 bootstrap resamples and adjustments mirroring those of Model III. All analytical processes were conducted utilizing R package version 3.4.3 and EmpowerStats version 4.1. A significance threshold of *P* < 0.05 guided the determination of statistical significance. Weight calculation for the 4 survey cycles was accomplished by dividing the MEC exam weights (WTMEC2YR) by 4.

## Results

### Baseline characteristics of study participants

The study enrolled a cohort of 1809 female participants, exhibiting an average age of 36.9 ± 7.6 years, and this cohort was categorized into two distinct groups based on self-reported GDM history. Among these women, 12.2% had a history of GDM. A comprehensive overview of the baseline characteristics is presented in Table 1. Women with a history of GDM were more likely to be obese (BMI ≥ 30 kg/m²), have diabetes and have delivered a baby of 9 pounds or more. Moreover, this group demonstrated a noteworthy reduction in lumbar BMD alongside a significantly elevated serum TC level in comparison to those without a GDM history.

**Table 1:** Characteristics of the study population

	No History of GDM (n = 1588)	History of GDM (n = 221)	*P* value
(Mean± SD)			
Age	37.01 ± 7.98	38.02 ± 6.79	0.0841
Lumbar BMD (g/cm^2^)	1.07 ± 0.14	1.04 ± 0.12	0.0113
25OHD (nmol/L)	64.73 ± 25.53	64.42 ± 22.44	0.8660
TC (mg/dL)	4.73 ± 0.91	4.96 ± 0.84	0.0006
[N (%)]			
Race			0.3673
Mexican American	204 (12.87)	36 (16.22)	
Other Hispanic	126 (7.95)	13 (6.06)	
Non-Hispanic White	898 (56.56)	124 (56.29)	
Non-Hispanic Black	215 (13.55)	23 (10.51)	
Other Race	144 (9.07)	24 (10.91)	
Education level			0.4785
< High school	260 (16.39)	41 (18.35)	
≥ High school	1328 (83.61)	180 (81.65)	
PIR			0.4634
< 1	340 (21.38)	52 (23.63)	
≥ 1	1248 (78.62)	169 (76.37)	
BMI (kg/m²)			< 0.0001
< 25	538 (33.89)	43 (19.25)	
≥ 25, < 30	447 (28.14)	56 (25.39)	
≥ 30	603 (37.97)	122 (55.36)	
Diabetes history			< 0.0001
No	1498 (94.31)	161 (72.98)	
Yes	90 (5.69)	60 (27.02)	
Hypertension history			0.6832
No	1331 (83.82)	183 (82.69)	
Yes	257 (16.18)	38 (17.31)	
Alcohol use			0.9367
Moderate	544 (34.27)	74 (33.40)	
Heavy	782 (49.24)	107 (48.39)	
No-drinking	195 (12.25)	30 (13.76)	
Unclear	67 (4.24)	10 (4.45)	
Smoking			0.4474
Current	340 (21.41)	46 (20.83)	
Former	233 (14.65)	40 (18.01)	
No-smoking	1015 (63.94)	135 (61.16)	
Delivered baby 9 lbs or more			< 0.0001
No	1366 (86.02)	167 (75.65)	
Yes	222 (13.98)	54 (24.35)	

GDM, gestational diabetes mellitus; BMD, bone mineral density; 25OHD, 25-hydroxyvitamin D; TC, total cholesterol; PIR, poverty income ratio; BMI, body mass index

### Results of multiple linear regression analysis

The results of the multiple linear regression analysis are shown in Table 2. We found a significant difference in lumbar BMD between respondents without and with a history of GDM (β=-0.026, 95% CI: -0.046, -0.006, *P* = 0.0113). The robustness of this discrepancy persisted when applying Model III, characterized by comprehensive adjustment for all relevant covariates (β=-0.023, 95% CI: -0.043, -0.003, *P* = 0.0275).

**Table 2:** Weighted linear regression results for relationship between GDM history and lumbar BMD.

	Model I β (95%CI) *P* value	Model II β (95%CI) *P* value	Model III β (95%CI) *P* value
No History of GDM	0	0	0
History of GDM	-0.026 (-0.046, -0.006) 0.0113	-0.024 (-0.044, -0.004) 0.0179	-0.023 (-0.043, -0.003) 0.0275

Model I adjust for: none

Model II adjust for: age; race

Model III adjust for: age; race; education level; PIR; BMI; diabetes history; hypertension history; alcohol use; smoking; 25OHD; delivered baby 9 lbs or more

GDM, gestational diabetes mellitus; BMD, bone mineral density; PIR, poverty income ratio; BMI, body mass index; 25OHD, 25-hydroxyvitamin D

Stratified analyses were thoughtfully employed, partitioning participants based on factors encompassing age, race, BMI, and a history of delivering a neonate weighing 9 pounds or more. As demonstrated in Table 3, subsequent to a comprehensive covariate adjustment, a noteworthy dissimilarity in lumbar BMD persisted between individuals without a history of GDM and those with such history in the subgroup categorized by age ≥ 35 years (β=-0.028, 95% CI: -0.053, -0.004, *P* = 0.0249), the non-Hispanic white population (β=-0.039, 95% CI: -0.074, -0.004, *P* = 0.0313), and those with a BMI ≥ 30 kg/m² (β=-0.034, 95% CI: -0.061, -0.008, *P* = 0.0116). However, the subgroup defined by “Delivered baby 9 lbs or more” exhibited no significant difference between the two subject groups.

**Table 3:** Stratified analyses of lumbar BMD in respondents, according to age, race, BMI, and delivered baby 9 lbs or more

	Model I β (95%CI) *P* value		Model II β (95%CI) *P* value		Model III β (95%CI) *P* value	
	No History of GDM	History of GDM	No History of GDM	History of GDM	No History of GDM	History of GDM
**Age**						
< 35y	0	-0.011 (-0.046, 0.023) 0.5317	0	-0.009 (-0.043, 0.025) 0.5991	0	-0.016 (-0.051, 0.019) 0.3827
≥35y	0	-0.034 (-0.060, -0.009) 0.0073	0	-0.030 (-0.055, -0.006) 0.0155	0	-0.028 (-0.053, -0.004) 0.0249
**Race**						
Mexican American	0	0.003 (-0.034, 0.040) 0.8908	0	0.002 (-0.035, 0.040) 0.9106	0	0.008 (-0.031, 0.048) 0.6824
Other Hispanic	0	0.053 (-0.006, 0.113) 0.0797	0	0.052 (-0.007, 0.112) 0.0856	0	0.058 (-0.010, 0.125) 0.0955
Non-Hispanic White	0	-0.042 (-0.077, -0.007) 0.0175	0	-0.042 (-0.077, -0.008) 0.0174	0	-0.039 (-0.074, -0.004) 0.0313
Non-Hispanic Black	0	0.025 (-0.026, 0.075) 0.3403	0	0.019 (-0.032, 0.070) 0.4618	0	0.027 (-0.024, 0.079) 0.3011
Other Race	0	-0.050 (-0.097, -0.003) 0.0382	0	-0.053 (-0.100, -0.006) 0.0295	0	-0.054 (-0.102, -0.007) 0.0251
**BMI**						
< 25 kg/m²	0	-0.047 (-0.097, 0.003) 0.0659	0	-0.043 (-0.092, 0.007) 0.0914	0	-0.045 (-0.093, 0.003) 0.0666
25–29 kg/m²	0	0.020 (-0.016, 0.057) 0.2682	0	0.020 (-0.016, 0.055) 0.2834	0	0.020 (-0.017, 0.057) 0.2929
≥30 kg/m²	0	-0.038 (-0.065, -0.011) 0.0063	0	-0.033 (-0.059, -0.006) 0.0150	0	-0.034 (-0.061, -0.008) 0.0116
**Delivered baby 9 lbs or more**						
No	0	-0.025 (-0.048, -0.002) 0.0352	0	-0.025 (-0.047, -0.002) 0.0338	0	-0.018 (-0.041, 0.005) 0.1253
Yes	0	-0.055 (-0.095, -0.015) 0.0073	0	-0.039 (-0.077, -0.000) 0.0482	0	-0.027 (-0.069, 0.015) 0.2115

Model I adjust for: none

Model II adjust for: age; race

Model III adjust for: age; race; education level; PIR; BMI; diabetes history; hypertension history; alcohol use; smoking; 25OHD; delivered baby 9 lbs or more. Stratification variables were not involved in the adjustment of the respective stratification analysis

GDM, gestational diabetes mellitus; BMD, bone mineral density; PIR, poverty income ratio; BMI, body mass index; 25OHD, 25-hydroxyvitamin D

Our analysis also encompassed a comprehensive examination of the relationship between TC and lumbar BMD, alongside the interrelationship between a historical occurrence of GDM and TC concentrations. Notably, Model III revealed a notable adverse association between TC and lumbar BMD (β=-0.008, 95% CI: -0.015, -0.001, *P* = 0.0318), as well as a significant difference in the level of TC between respondents without and with a history of GDM (β = 0.163, 95% CI: 0.033, 0.293, *P* = 0.0142) (Table 4).

**Table 4:** Weighted linear regression results for relationship between TC and lumbar BMD, GDM history and TC.

	Model I β (95%CI) *P* value	Model II β (95%CI) *P* value	Model III β (95%CI) *P* value
**TC and lumbar BMD**			
Total Cholesterol (mg/dL)	-0.008 (-0.015, -0.001) 0.0289	-0.008 (-0.015, -0.001) 0.0228	-0.008 (-0.015, -0.001) 0.0318
**GDM history and TC**			
No History of GDM	0	0	0
History of GDM	0.230 (0.099, 0.362) 0.0006	0.196 (0.068, 0.323) 0.0027	0.163 (0.033, 0.293) 0.0142

Model I adjust for: none

Model II adjust for: age; race

Model III adjust for: age; race; education level; PIR; BMI; diabetes history; hypertension history; alcohol use; smoking; 25OHD; delivered baby 9 lbs or more

TC, total cholesterol; GDM, gestational diabetes mellitus; BMD, bone mineral density; PIR, poverty income ratio; BMI, body mass index; 25OHD, 25-hydroxyvitamin D

### Mediation analysis

**Fig. 2:**
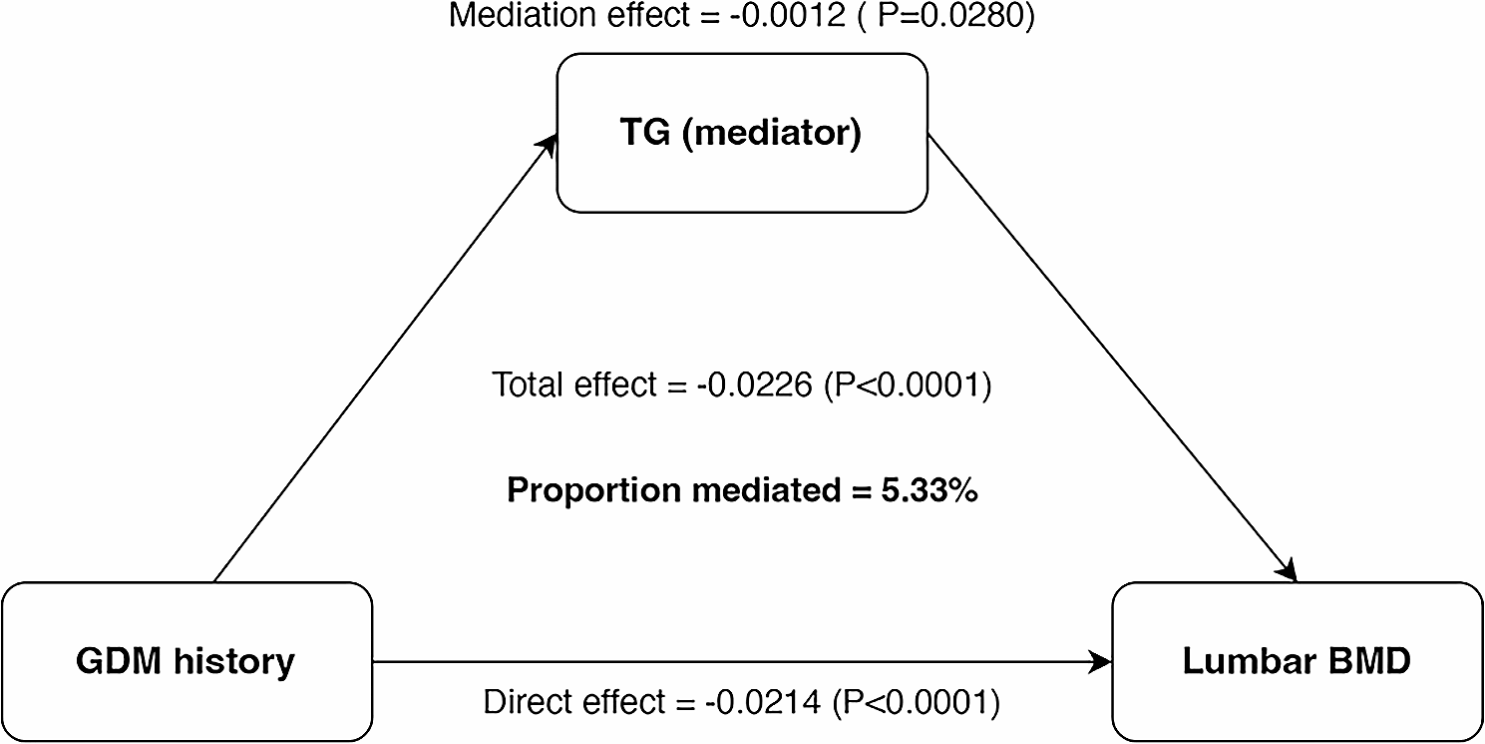
Effect of the TC (mediators) on the relationship between a history of GDM (exposure) and lumbar BMD (outcome). TC, total cholesterol; GDM, gestational diabetes mellitus; BMD, bone mineral density

Mediation analyses were executed to quantify the degree to which TC served as a mediator in the relationship linking a history of GDM with lumbar BMD. Following adjustments encompassed in Model III, our investigation unveiled a compelling revelation: GDM history had a significant direct effect on lumbar BMD (*P* < 0.0001), whereas TC partially mediated the mediation effect of GDM history on lumbar BMD (*P* = 0.0280). Importantly, our estimations indicate that TC elucidates 5.33% of the multifaceted association connecting GDM history and lumbar BMD, visually depicted in Fig. 2.

## Discussion

The study conducted a cross-sectional analysis utilizing data extracted from the NHANES database spanning 2011 to 2018, comprising a cohort of 1809 women aged 20 to 49 years. We investigated the association between the GDM history and lumbar BMD, while concurrently assessing the potential mediating function attributed to total cholesterol. Upon executing comprehensive adjustments, our findings unveiled those women with a history of GDM exhibited a 2.6% reduction in lumbar BMD compared to those without such history. An evident positive association was discerned between the GDM history and TC levels, whereas a negative association was observed between TC and lumbar BMD. Notably, TC exhibited partial mediation, contributing to the relationship between the GDM history and lumbar BMD.

The state of pregnancy induces substantial physiological changes in the maternal organism. Concurrently, pregnant women experience heightened susceptibility to bone loss, a phenomenon attributed to the physiological utilization of maternal bone mass for fetal skeletal development. This process may be exacerbated by disturbances in glucose metabolism during pregnancy [20]. In the investigation conducted by Wong et al. [21], BMD changes in pregnant women with GDM were assessed using quantitative ultrasonographic measurements of the Achilles bone. The findings indicated higher BMD loss in women with GDM compared to those without GDM. Additionally, Han et al. [22] employed ultrasound bone densitometry in conjunction with vitamin D assessment to evaluate BMD in GDM patients. The study revealed lower BMD and 25-hydroxyvitamin levels in GDM patients compared to normal pregnant women.

While some individuals with GDM may experience natural regression post-delivery, it cannot be ignored that that women with a GDM history exhibit a heightened likelihood of developing T2DM. A systematic review and meta-analysis have revealed an aggregate prevalence of T2DM reaching 16.46% within this cohort, alongside a concomitant 9.91% prevalence evident in Caucasian women [23]. T2DM is a risk factor for osteoporosis, and in contrast to non-diabetic patients, diabetic Patients have an increased relative risk (RR) of fracture compared to non-diabetic patients [24, 25]. However, multiple investigations have discerned that individuals afflicted with T2DM frequently manifest normal or elevated levels of bone mineral density [26–28]. Plausible rationales for this disparity emerge from structural modifications within bone microarchitecture induced by diabetes, entailing deviations in osteoblastic function, matrix composition, osteoclastic apoptosis, osteoclastic differentiation, and osteoclast-mediated bone resorptive processes. Collectively, these disturbances culminate in bone material of heightened fragility, rendering it predisposed to fractures in the presence of reduced mechanical loading or compromised biomechanical attributes [29, 30].

Age-related alterations in women’s estrogen levels contribute to accelerated bone turnover and subsequent bone loss [31]. To minimize the potential confounding influence of estrogen levels, women over the age of 50 and estrogen users were excluded from our study cohort, focusing the analysis on the association between a history of GDM and women’s bone health. Notably, bone growth reaches its maximum and strongest size between the ages of 30 to 35 years, followed by a decline in bone mineral content after 35 years [32]. Accordingly, our subgroup analysis utilized a cutoff age of 35 years, revealing a 2.8% lower lumbar bone mineral density (BMD) in women aged ≥ 35 years with a history of GDM compared to those without such a history in a fully adjusted model. This finding may suggest that women aged ≥ 35 years with a history of GDM should be more concerned about bone health. Furthermore, in the BMI subgroup, women with a previous history of gestational diabetes and a BMI ≥ 30 kg/m² exhibited a notable reduction of 3.4% in lumbar BMD contrast to their counterparts lacking such medical history. It has been demonstrated that women with obesity exhibit a diminished relative BMD compared to women with normal weight, potentially heightening the susceptibility to fractures [33]. Intriguingly, some studies have advanced the concept that obesity may impart a protective effect against osteoporosis in middle-aged and elderly women [34, 35].

GDM is associated with an increased risk of dyslipidemia in women postpartum. Studies indicate that individuals with GDM and those with a history of GDM exhibit significant abnormalities in their lipid profile compared to the healthy population, including elevated serum total cholesterol levels [36, 37]. Insulin resistance is one of the pathogenic mechanisms of GDM and contributes to dyslipidemia in pregnant women with GDM and in the postpartum period [38]. Dyslipidemia is also significantly associated with bone health in women, but most of such studies have focused on postmenopausal women [39, 40]. Notably, our results showed that there was still a negative correlation between TC and lumbar BMD in the premenopausal female population, and mediation analysis revealed that TC partially mediated the correlation between a history of prior gestational diabetes and lumbar BMD. This phenomenon may be due to the fact that elevated cholesterol inhibits osteoblast differentiation while enhancing osteoclast production and activity [41]. These interconnected processes collectively contribute to a decrease in bone mineral density. Considering this relationship, premenopausal women with a history of GDM may have the opportunity to modulate their cholesterol levels through dietary or lifestyle interventions, thereby enhancing bone health and averting prospective osteoporosis.

In this study, our attention was not only directed towards investigating the association between a history of GDM and lumbar BMD in the premenopausal female population but also extended to the exploration of potential mechanisms through mediation analyses. These analyses, to a certain extent, yield insights into strategies for enhancing the skeletal health of this demographic. Several noteworthy limitations warrant consideration in this study. Firstly, the cross-sectional design of our study prevents the determination of a causal relationship between history of GDM and lumbar BMD, and further validation through prospective cohort studies or randomized controlled trials is necessary. Secondly, it is crucial to recognize that unmeasured confounding variables may continue to exert influence on the association between a history of GDM and lumbar BMD. Despite the indispensable role of mediation analysis, it is vital to emphasize that the results within the cross-sectional framework persistently maintain a correlative nature and lack causality. In addition, GDM history was based on self-report and misclassification may be of concern. Nevertheless, data from NHANES are considered valid for assessing the prevalence of GDM in the general population [42–44]. Finally, because the current study focused on women aged 20–49 years in the United States, the results cannot be generalized to all age groups.

## Conclusion

Among women aged 20–49 with GDM history, lumbar BMD is reduced compared to non-GDM peers. Total cholesterol is an important moderator of the observed relationship between a history of GDM and lumbar BMD. Prospective and mechanistic studies are needed to validate and extend these findings.

## Data Availability

All data for this study were available at the National Health and Nutrition Examination Survey (NHANES) website https://www.cdc.gov/Nchs/Nhanes/about_nhanes.htm.

## Abbreviations

GDM: gestational diabetes mellitus
BMD: bone mineral density
TC: total cholesterol
HAPO: Hyperglycemia and Adverse Pregnancy Outcomes
IADPSG: International Association of the Diabetes and Pregnancy Study Groups
T2DM: Type 2 diabetes mellitus
NHANES: National Health and Nutrition Examination Surveys
DXA: dual-energy X-ray absorptiometry
BMI: body mass index
PIR: poverty-to-income ratio
25OHD: 25-hydroxyvitamin D
RR: relative risk

## References

[1] American Diabetes Association (2020). )2. Classification and diagnosis of Diabetes: standards of Medical Care in Diabetes-2020. Diabetes Care.

[2] Sweeting A, Wong J, Murphy HR, Ross GP (2022). A clinical update on gestational Diabetes Mellitus. Endocr Rev.

[3] Casagrande SS, Linder B, Cowie CC (2018). Prevalence of gestational Diabetes and subsequent type 2 Diabetes among U.S. women. Diabetes Res Clin Pract.

[4] Carr DB, Utzschneider KM, Hull RL (2006). Gestational Diabetes Mellitus increases the risk of Cardiovascular Disease in women with a family history of type 2 Diabetes. Diabetes Care.

[5] Retnakaran R, Qi Y, Connelly PW, Sermer M, Hanley AJ, Zinman B (2010). The graded relationship between glucose tolerance status in pregnancy and postpartum levels of low-density-lipoprotein cholesterol and apolipoprotein B in young women: implications for future cardiovascular risk. J Clin Endocrinol Metab.

[6] Gunderson EP, Chiang V, Pletcher MJ, Jacobs DR, Quesenberry CP, Sidney S, Lewis CE (2014). History of gestational Diabetes Mellitus and future risk of Atherosclerosis in mid-life: the coronary artery risk development in young adults study. J Am Heart Assoc.

[7] Metzger BE, Lowe LP, HAPO Study Cooperative Research Group (2008). Hyperglycemia and adverse pregnancy outcomes. N Engl J Med.

[8] Bianco ME, Josefson JL (2019). Hyperglycemia during pregnancy and long-term offspring outcomes. Curr Diab Rep.

[9] Compston JE, McClung MR, Leslie WD (2019). Osteoporos Lancet.

[10] Shanbhogue VV, Mitchell DM, Rosen CJ, Bouxsein ML (2016). Type 2 Diabetes and the skeleton: new insights into sweet bones. Lancet Diabetes Endocrinol.

[11] Dede AD, Tournis S, Dontas I, Trovas G (2014). Type 2 Diabetes Mellitus and fracture risk. Metabolism.

[12] Makovey J, Chen JS, Hayward C, Williams FMK, Sambrook PN (2009). Association between serum cholesterol and bone mineral density. Bone.

[13] Ahmeidat A, Bhattacharya S, Luben RN, Khaw K-T, Myint PK (2021). Long-term effects of gestational Diabetes on bone mineral density and fracture risk: analysis of the Norfolk cohort of the European prospective investigation into Cancer (EPIC-Norfolk) population-based study. Maturitas.

[14] Lu B, Zhang L (2023). Association of a history of gestational Diabetes Mellitus with osteoporosis, bone mineral density, and trabecular bone score in postmenopausal women. Diabetol Metab Syndr.

[15] Appiah D, Nwabuo CC, Ebong IA, Wellons MF, Winters SJ (2021). Trends in Age at Natural Menopause and Reproductive Life Span among US women, 1959–2018. JAMA.

[16] Curtin LR, Mohadjer LK, Dohrmann SM, Montaquila JM, Kruszan-Moran D, Mirel LB, Carroll MD, Hirsch R, Schober S, Johnson CL (2012). The National Health and Nutrition Examination Survey: Sample Design, 1999–2006. Vital Health Stat.

[17] Kanis JA, Johnell O (2005). Requirements for DXA for the management of osteoporosis in Europe. Osteoporos Int.

[18] Tofighi D, MacKinnon DP (2011). RMediation: an R package for mediation analysis confidence intervals. Behav Res Methods.

[19] Valeri L, Vanderweele TJ (2013). Mediation analysis allowing for exposure-mediator interactions and causal interpretation: theoretical assumptions and implementation with SAS and SPSS macros. Psychol Methods.

[20] Namgung R, Tsang RC (2003). Bone in the pregnant mother and newborn at birth. Clin Chim Acta.

[21] To WWK, Wong MWN (2008). Bone mineral density changes in gestational diabetic pregnancies-a longitudinal study using quantitative ultrasound measurements of the os calcis. Gynecol Endocrinol.

[22] Han L, Ma J, Wang S, Li Z (2022). Evaluation of bone mineral density in patients with gestational Diabetes Mellitus by ultrasonic bone mineral density measurement combined with Vitamin-D deficiency and analysis of influencing factors. Pak J Med Sci.

[23] Vounzoulaki E, Khunti K, Abner SC, Tan BK, Davies MJ, Gillies CL (2020). Progression to type 2 Diabetes in women with a known history of gestational Diabetes: systematic review and meta-analysis. BMJ.

[24] Jackuliak P, Payer J (2014). Osteoporosis, fractures, and Diabetes. Int J Endocrinol.

[25] Liao C-C, Lin C-S, Shih C-C, Yeh C-C, Chang Y-C, Lee Y-W, Chen T-L (2014). Increased risk of fracture and postfracture adverse events in patients with Diabetes: two Nationwide Population-based Retrospective Cohort studies. Diabetes Care.

[26] Cortet B, Lucas S, Legroux-Gerot I, Penel G, Chauveau C, Paccou J (2019). Bone disorders associated with Diabetes Mellitus and its treatments. Joint Bone Spine.

[27] Oei L, Zillikens MC, Dehghan A (2013). High bone mineral density and fracture risk in type 2 Diabetes as skeletal Complications of inadequate glucose control: the Rotterdam Study. Diabetes Care.

[28] Jang M, Kim H, Lea S, Oh S, Kim JS, Oh B (2018). Effect of duration of Diabetes on bone mineral density: a population study on east Asian males. BMC Endocr Disord.

[29] De Liefde II, Van Der Klift M, De Laet CEDH, Van Daele PLA, Hofman A, Pols HAP (2005). Bone mineral density and fracture risk in type-2 Diabetes Mellitus: the Rotterdam Study. Osteoporos Int.

[30] Murray C (2019). Impact of Diabetes Mellitus on Bone Health. IJMS.

[31] Kanis JA, Cooper C, Rizzoli R, Reginster J-Y, Scientific Advisory Board of the European Society for Clinical and Economic Aspects of Osteoporosis (ESCEO) and the Committees of Scientific Advisors and National Societies of the International Osteoporosis Foundation (IOF) (2019). European guidance for the diagnosis and management of osteoporosis in postmenopausal women. Osteoporos Int.

[32] Al-Bogami MM, Akanle OA, Aldawood S, Alkhorayef M, Sulieman A, Jawad AS, Mageed RA (2023). Comparison of bone mineral density changes between male and female osteoporosis patients using dual energy X-ray absorptiometry scan. Appl Radiat Isot.

[33] Rudman HA, Birrell F, Pearce MS, Tuck SP, Francis RM, Treadgold L, Hind K (2019). Obesity, bone density relative to body weight and prevalent vertebral fracture at age 62 years: the Newcastle thousand families study. Osteoporos Int.

[34] Turcotte A-F, O’Connor S, Morin SN, Gibbs JC, Willie BM, Jean S, Gagnon C (2021). Association between obesity and risk of fracture, bone mineral density and bone quality in adults: a systematic review and meta-analysis. PLoS ONE.

[35] Morin S, Tsang JF, Leslie WD (2009). Weight and body mass index predict bone mineral density and fractures in women aged 40 to 59 years. Osteoporos Int.

[36] Chodick G, Tenne Y, Barer Y, Shalev V, Elchalal U (2020). Gestational Diabetes and long-term risk for dyslipidemia: a population-based historical cohort study. BMJ Open Diabetes Res Care.

[37] Sánchez-Vera I, Bonet B, Viana M, Quintanar A, Martín MD, Blanco P, Donnay S, Albi M (2007). Changes in plasma lipids and increased low-density lipoprotein susceptibility to oxidation in pregnancies complicated by gestational Diabetes: consequences of obesity. Metabolism.

[38] Herrera E, Desoye G (2016). Maternal and fetal lipid metabolism under normal and gestational diabetic conditions. Horm Mol Biol Clin Investig.

[39] Zhang Q, Zhou J, Wang Q, Lu C, Xu Y, Cao H, Xie X, Wu X, Li J, Chen D (2020). Association between Bone Mineral Density and lipid Profile in Chinese women. Clin Interv Aging.

[40] Chen Y-Y, Wang W-W, Yang L, Chen W-W, Zhang H-X (2018). Association between lipid profiles and osteoporosis in postmenopausal women: a meta-analysis. Eur Rev Med Pharmacol Sci.

[41] Pelton K, Krieder J, Joiner D, Freeman MR, Goldstein SA, Solomon KR (2012). Hypercholesterolemia promotes an osteoporotic phenotype. Am J Pathol.

[42] Mao Y, Hu W, Liu L, Liu Q (2022). Association between Gestational Diabetes Mellitus and Future risk of kidney stones. Front Public Health.

[43] Dorans KS, Bazzano LA, Li X, Bundy JD, Tian L, He J (2022). Lifestyle behaviors and cardiovascular risk profiles among parous women by gestational Diabetes status, 2007–2018. Nutr Metab Cardiovasc Dis.

[44] Xiao RS, Simas TAM, Person SD, Goldberg RJ, Waring ME (2015). Diet quality and history of gestational Diabetes Mellitus among childbearing women, United States, 2007–2010. Prev Chronic Dis.

